# Suppression of RNAi by dsRNA-Degrading RNaseIII Enzymes of Viruses in Animals and Plants

**DOI:** 10.1371/journal.ppat.1004711

**Published:** 2015-03-06

**Authors:** Isabel Weinheimer, Yaming Jiu, Minna-Liisa Rajamäki, Olli Matilainen, Jukka Kallijärvi, Wilmer J. Cuellar, Rui Lu, Mart Saarma, Carina I. Holmberg, Jussi Jäntti, Jari P. T. Valkonen

**Affiliations:** 1 Department of Agricultural Sciences, University of Helsinki, Helsinki, Finland; 2 Institute of Biotechnology, University of Helsinki, Helsinki, Finland; 3 Research Programs Unit, Translational Cancer Biology, and Institute of Biomedicine, Biomedicum Helsinki, University of Helsinki, Helsinki, Finland; 4 Department of Biological Sciences, Louisiana State University, Baton Rouge, Louisiana, United States of America; 5 VTT Technical Research Centre of Finland, Espoo, Finland; Institute of Microbiology of the Chinese Academy of Sciences, CHINA

## Abstract

Certain RNA and DNA viruses that infect plants, insects, fish or poikilothermic animals encode Class 1 RNaseIII endoribonuclease-like proteins. dsRNA-specific endoribonuclease activity of the RNaseIII of rock bream iridovirus infecting fish and Sweet potato chlorotic stunt crinivirus (SPCSV) infecting plants has been shown. Suppression of the host antiviral RNA interference (RNAi) pathway has been documented with the RNaseIII of SPCSV and Heliothis virescens ascovirus infecting insects. Suppression of RNAi by the viral RNaseIIIs in non-host organisms of different kingdoms is not known. Here we expressed PPR3, the RNaseIII of Pike-perch iridovirus, in the non-hosts *Nicotiana benthamiana* (plant) and *Caenorhabditis elegans* (nematode) and found that it cleaves double-stranded small interfering RNA (ds-siRNA) molecules that are pivotal in the host RNA interference (RNAi) pathway and thereby suppresses RNAi in non-host tissues. In *N*. *benthamiana*, PPR3 enhanced accumulation of Tobacco rattle tobravirus RNA1 replicon lacking the 16K RNAi suppressor. Furthermore, PPR3 suppressed single-stranded RNA (ssRNA)—mediated RNAi and rescued replication of Flock House virus RNA1 replicon lacking the B2 RNAi suppressor in *C*. *elegans*. Suppression of RNAi was debilitated with the catalytically compromised mutant PPR3-Ala. However, the RNaseIII (CSR3) produced by SPCSV, which cleaves ds-siRNA and counteracts antiviral RNAi in plants, failed to suppress ssRNA-mediated RNAi in *C*. *elegans*. In leaves of *N*. *benthamiana*, PPR3 suppressed RNAi induced by ssRNA and dsRNA and reversed silencing; CSR3, however, suppressed only RNAi induced by ssRNA and was unable to reverse silencing. Neither PPR3 nor CSR3 suppressed antisense-mediated RNAi in *Drosophila melanogaster*. These results show that the RNaseIII enzymes of RNA and DNA viruses suppress RNAi, which requires catalytic activities of RNaseIII. In contrast to other viral silencing suppression proteins, the RNaseIII enzymes are homologous in unrelated RNA and DNA viruses and can be detected in viral genomes using gene modeling and protein structure prediction programs.

## Author Summary

RNA interference (RNAi) is a cellular mechanism activated by double-stranded RNA (dsRNA). Cellular dsRNA-specific RNaseIII enzymes (Dicer) recognize dsRNA and process it into double-stranded small interfering RNAs (ds-siRNAs) of 21–25 nucleotides (nt). siRNAs guide RNAi to degrade also single-stranded RNA homologous to the trigger. RNAi regulates gene expression, controls transposons, and represents an important antiviral defense mechanism. Therefore, viruses encode proteins dedicated to countering RNAi. In this study, the RNaseIII enzymes of a fish DNA virus (PPIV) and a plant RNA virus (SPCSV) were compared for suppression of RNAi in non-host organisms. The fish iridovirus RNaseIII suppressed RNAi in a plant and a nematode. It also enhanced accumulation of an RNAi suppressor deficient virus in plants, and suppressed antiviral RNAi and could rescue multiplication of an unrelated, RNAi suppressor-defective virus in nematodes. In contrast, the plant virus RNaseIII could suppress RNAi only in plants. Our results underscore that the active viral RNaseIII enzymes suppress RNAi. Their activity in suppression of RNAi seems to differ for the spectrum of unrelated organisms. Understanding of this novel mechanism of RNAi suppression may inform means of controlling the diseases and economic losses which the RNaseIII-containing viruses cause in animal and plant production.

## Introduction

Eukaryotic RNA interference (RNAi) pathways are activated by double-stranded RNA (dsRNA) and function in gene regulation and antiviral defense [1–5]. In invertebrates, genes can be silenced via dsRNA as demonstrated in the nematode *Caenorhabditis elegans* [6] and the fruit fly *Drosophila melanogaster* [7], whereas in plants post-transcriptional gene silencing also can be induced by homologous antisense or positive-sense single-stranded RNA (ssRNA) [8]. Induction of sense-mediated RNAi typically requires the activity of a cellular RNA-dependent RNA polymerase (RdRp) for synthesis of dsRNA on the sense RNA transcript [9]. Class 3 RNaseIII endoribonucleases known as Dicers contain a dsRNA-binding domain, two catalytic domains (RNaseIII signature motifs), an N-terminal helicase, and a PAZ domain [10, 11]. Dicers recognize dsRNA and process it into double-stranded small interfering RNAs (ds-siRNAs) that are 21–25 nucleotides (nt) long [1, 12]. siRNAs bind to and guide the cellular RNase AGO to cleave complementary ssRNA molecules [13, 14]. RdRp helps to amplify RNAi via production of secondary triggers of RNAi derived from cleaved RNA in plants and nematodes (*C*. *elegans*) [12, 15] and hence also contributes to the generation of secondary siRNAs acting as mobile signals for systemic RNAi in plants, nematodes, and possibly insects (*D*. *melanogaster*) [5, 16].

Replicating viruses are vulnerable to RNAi, because the double-stranded replicative intermediates of viral RNA genomes, the secondary structures of RNA transcripts, and the sense-antisense transcript pairs resulting from bidirectional transcription of DNA viruses can be recognized by Dicers. Therefore, viruses encode proteins dedicated to countering RNAi and protecting viral RNA from degradation. Viral RNAi suppressor proteins interfere with the host antiviral RNAi pathway, for example by binding to the dicing complex, dsRNA or siRNA, by preventing assembly of the AGO-containing silencing complex, or inhibiting production of secondary ds-siRNA [16, 17].

The viral RNAi suppressor proteins identified to date do not contain conserved amino acid motifs or other structural features, but a variety of different types of viral proteins can suppress RNAi and target the same molecular components and steps in the RNAi pathway [17, 18]. It is therefore not possible to recognize an RNAi suppressor without carrying out pertinent experiments. However, certain plant and animal viruses encode homologous dsRNA-specific Class 1 RNaseIII enzymes [19–22], of which the dsRNA-specific Class 1 RNaseIII endoribonuclease termed RNase3 (designated as CSR3 in this paper) of *Sweet potato chlorotic stunt virus* (SPCSV) suppresses RNAi [21]. SPCSV contains a positive-sense ssRNA [(+)ssRNA] genome, but also iridoviruses (family *Iridoviridae*) which have large dsDNA genomes and infect invertebrate or poikilothermic vertebrate animals [19], encode putative Class 1 RNaseIII enzymes. Indeed, the RNaseIII of rock bream iridovirus is a dsRNA-specific endoribonuclease [20], but little is known about its role in infection and suppression of RNAi. *Heliothis virescens ascovirus 3e* (HvAV-3e, family *Ascoviridae*) contains a DNA genome and infects insects. It encodes an RNaseIII that was shown to suppress gene silencing following expression from a recombinant baculovirus in infected insect cells [22].

**Fig 1:**
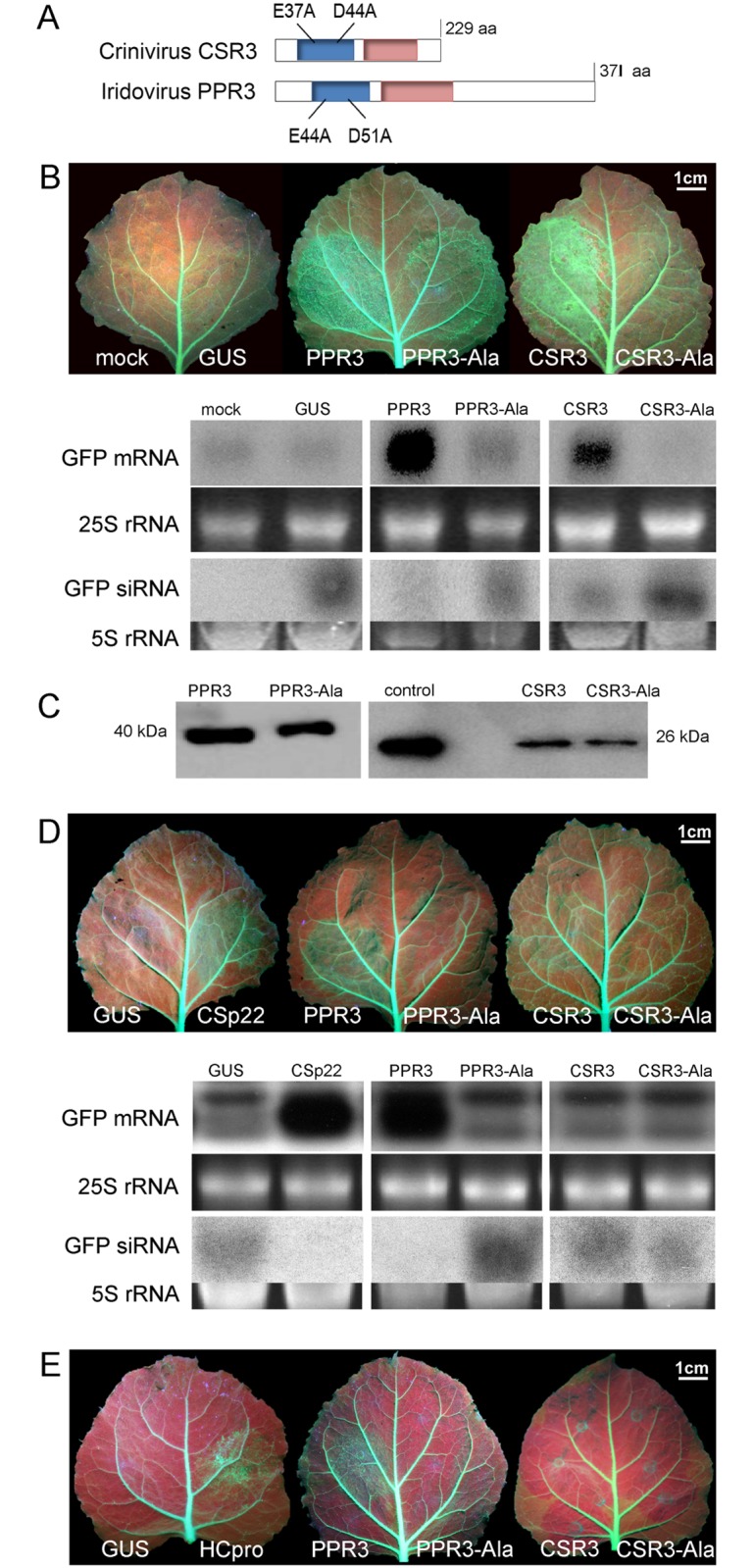
Class 1 RNaseIII endoribonucleases of PPIV (PPR3) and SPCSV (CSR3) suppress RNAi in leaves of *Nicotiana benthamiana*. (**A**) The RNaseIII signature motif (blue) and dsRNA-binding domain (red) conserved in Class 1 RNaseIII endoribonucleases, and substitution of two conserved amino acid residues of the catalytic site are depicted (mutated proteins PPR3-Ala and CSR3-Ala). (**B**) Suppression of sense ssRNA—induced RNAi. The left and right side of leaves of *gfp*-transgenic *N*. *benthamiana* line 16c were infiltrated with *Agrobacterium tumefaciens* (Agro) strains expressing *gfp* to induce silencing of the constitutively expressed *gfp* transgene (note green fluorescence in leaf veins) and co-infiltrated with Agro strains for expression of PPR3, PPR3-Ala, CSR3, CSR3-Ala, or GUS (β-glucuronidase, negative control) or mock-infiltrated with buffer (negative control). Leaves were photographed and analyzed 3 days post-infiltration. Accumulation of *gfp* mRNA and siRNA was analyzed by northern blotting. Ethidium bromide—stained gels of rRNA were used as loading controls. (**C**) Immunoblot of the RNaseIII proteins in the infiltrated tissues shown in (B). Control indicates purified recombinant CSR3 (positive control). (**D**) Suppression of dsRNA (hairpin RNA)-induced RNAi. Co-infiltration was carried out as in (B), except that an Agro strain expressing double-stranded (hairpin) *gfp* was used. Agro strains expressing GUS or the SPCSV p22 silencing suppressor were used as a negative and a positive control, respectively. Accumulation of *gfp* mRNA and siRNA was determined by northern blotting. (**E**) Reversion of RNAi. Suppression of the *gfp* transgene was achieved by sense-mediated silencing as in (B). After 24 h, the same leaf spots were infiltrated with an Agro strain for expression of PPR3, PPR3-Ala, CSR3, CSR3-Ala, GUS (negative control), or the HCpro silencing suppressor of *Potato virus A* (positive control) and photographed 3 days later.

Compared with Dicers, Class 1 RNaseIIIs have a simple structure. Similar to the Class 1 RNaseIII of *Escherichia coli* [23], CSR3 contains a single catalytic domain and a dsRNA-binding domain and cleaves long dsRNA molecules in an Mg^2+^-dependent manner [21]. CSR3 cleaves ds-siRNA, suppresses sense-mediated RNAi, and counteracts antiviral RNAi in plants [24]. The RNAseIII of HvAV-3e also cleaves ds-siRNA [22]. However, it is not known whether the iridovirus RNaseIII can suppress RNAi, and therefore we compared RNAi suppression potential between the *Pike-perch iridovirus* (PPIV) Class 1 RNaseIII (PPR3) and CSR3 in plant and animal tissues (Fig. 1A). We were also interested to find out whether these proteins have broad spectrum of activity allowing suppression of RNAi in both animal and plant kingdoms. Our results reveal that the viral Class 1 RNaseIII enzymes have conserved functions in RNAi suppression, making it possible to identify this class of RNA suppressors using bioinformatics approaches, but the spectrum of unrelated organisms in which they are active differs.

## Results

### Suppression of RNAi by the Viral RNaseIII Enzymes PPR3 and CSR3 in Plants

The ability of PPR3 to suppress sense-mediated RNAi in *Nicotiana benthamiana* was tested using an agroinfiltration assay of leaves of transgenic *N*. *benthamiana* (line 16c) that constitutively expressed the jellyfish green fluorescent protein (GFP) under the *Cauliflower mosaic virus* 35S promoter [24–26]. Leaves were co-infiltrated with a liquid culture of *Agrobacterium tumefaciens* engineered with a 35S-GFP transgene and *A*. *tumefaciens* expressing 35S promoter—driven PPR3, CSR3, or GUS (β-glucuronidase; negative control). Consequently, GFP fluorescence and *gfp* mRNA level were initially enhanced but decreased substantially to the level of the constitutive expression of the *gfp* transgene in the leaves co-infiltrated to express GFP and GUS, as expected and consistent with sense-mediated silencing of *gfp* (Fig. 1B). In contrast, *gfp* mRNA level and GFP fluorescence increased and remained high by 3 days post-infiltration (d.p.i.) in leaf tissues co-infiltrated to overexpress GFP and PPR3 or CSR3 (Fig. 1B), consistent with suppression of *gfp* silencing. The accumulation of *gfp* mRNA-derived siRNA correlated inversely with *gfp* mRNA accumulation, as expected (Fig. 1B).

**Fig 2:**
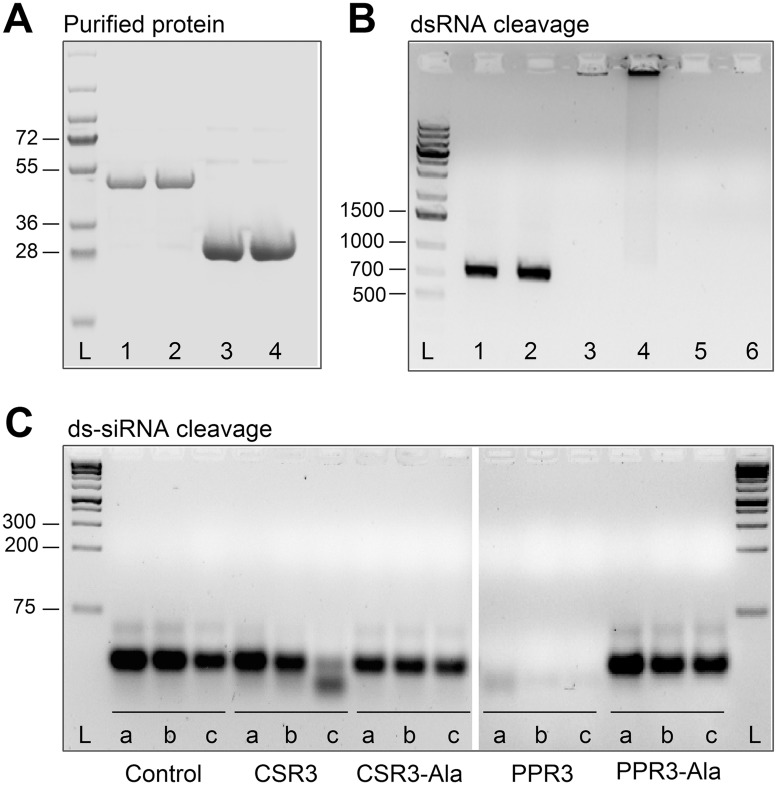
Endoribonuclease activity of PPR3 and CSR3 on long and short dsRNA. (**A**) His-tagged proteins PPR3 (lane 1), PPR3-Ala (lane 2), CSR3 (lane 3), and CSR3-Ala (lane 4) expressed in *E*. *coli* and analyzed by SDS-PAGE. L, Prestained protein ladder (Fermentas). Expected sizes of PPR3 and PPR3-ala **~**40 kDa; CSR3 and CSR3-Ala **~**26 kDa. (**B**) CSR3 (lane 3), PPR3 (lane 5), and PPR3-Ala (lane 6) processed long dsRNA (700 bp) following incubation at 37°C for 30 min, as detected by agarose gel electrophoresis, whereas CSR3-Ala (lane 4) bound dsRNA but was not able to process it (dsRNA remained in the well of the gel). The eluate (lane 2) and the reaction buffer (lane 1) were used as negative controls. Results (lane 3) indicate that the amount of dsRNA used in the reaction exceeded the catalytic activity of CSR3 under the prevailing experimental conditions and the unprocessed portion of dsRNA bound but not processed by CSR3 remained in the well (lane 3). Furthermore, the results indicated that the mutations introduced to PPR3 did not fully abolish the catalytic activities on long dsRNA. L, 1-kb DNA size marker. (**C**) Endoribonuclease activity of 0.5 μM CSR3, PPR3, CSR3-Ala, and PPR3-Ala on synthetic double-stranded siRNA (24 bp) in a 3-h incubation at 37°C at pH 6.5 (a), 7.5 (b), or 8.5 (c). Samples were analyzed by electrophoresis using a 4% agarose gel.

The endoribonuclease signature motif of Class I RNaseIII enzymes is conserved, and the structure of the catalytic domain of *E*. *coli* Class 1 RNaseIII and the amino acid residues critical for catalytic activity have been elucidated [22]. We have shown that when the corresponding critical residues are replaced with alanine in CSR3 (E37A and D44A; mutant CSR3-Ala), the RNaseIII and RNAi suppression activities of CSR3 are abolished [24]. In the current study, the corresponding mutations (E44A and D51A) were introduced to the endoribonuclease signature motif of PPR3 to yield the mutant PPR3-Ala (Fig. 1A). PPR3, PPR3-Ala, CSR3, and CSR3-Ala were expressed in *E*. *coli*, and the His-tagged recombinant proteins were purified (Fig. 2A). CSR3 and PPR3 processed long dsRNA (Fig. 2B, lanes 3 and 5, respectively) and synthetic ds-siRNA *in vitro* (Fig. 2C). While PPR3-Ala retained endoribonuclease activity for long dsRNA despite of the two mutations (Fig. 2B, lane 6), it failed to cleave ds-siRNA *in vitro* (Fig. 2C). In contrast, CSR3-Ala could not process either long dsRNA (Fig. 2B, lane 4) or ds-siRNA (Fig. 2C) as previously [24]. When GFP was co-expressed with CSR3-Ala or PPR3-Ala in leaves of *N*. *benthamiana* 16c as above, only constitutive or slightly higher expression levels, respectively, were observed at 3 d.p.i. (Fig. 1B), indicating that the endoribonuclease activities of CSR3 and PPR3 were needed to protect *gfp* mRNA from degradation. Results with PPR3 suggested that cleavage of ds-siRNA was particularly important for suppression of RNAi.

PPR3 and CSR3 were tested for suppression of dsRNA (hairpin RNA)-induced gene silencing. The agroinfiltration assay was carried out as above, except that an *A*. *tumefaciens* strain expressing hairpin-*gfp* RNA was used instead of GFP. PPR3 was able to suppress dsRNA-induced silencing of *gfp*, similar to the p22 RNAi suppressor protein of SPCSV [21] (Fig. 1D). In contrast, PPR3-Ala, CSR3, and CSR3-Ala failed to interfere with dsRNA-mediated silencing (Fig. 1D).

Certain viral RNAi suppressors cannot reverse RNAi after RNAi is initiated [18]. To test this aspect with PPR3 and CSR3, sense-mediated silencing of *gfp* was induced in leaves of *N*. *benthamiana* line 16c by agroinfiltration as above. After 24 h, the same leaves were agroinfiltrated for expression of PPR3, PPR3-Ala, CSR3, or CSR3-Ala. Expression of PPR3 enhanced GFP fluorescence in the leaves at 3 d.p.i., similar to the helper component proteinase (HCpro) RNAi suppressor of plant potyviruses (family *Potyviridae*) (Fig. 1E) known to reverse silencing [25]. In contrast, PPR3-Ala, CSR3, and CSR3-Ala failed to reverse *gfp* silencing (Fig. 1E).

### PPR3, Unlike CSR3, Suppresses RNAi in *C*. *elegans*, but Neither Protein Suppresses RNAi in *Drosophila melanogaster*

**Fig 3:**
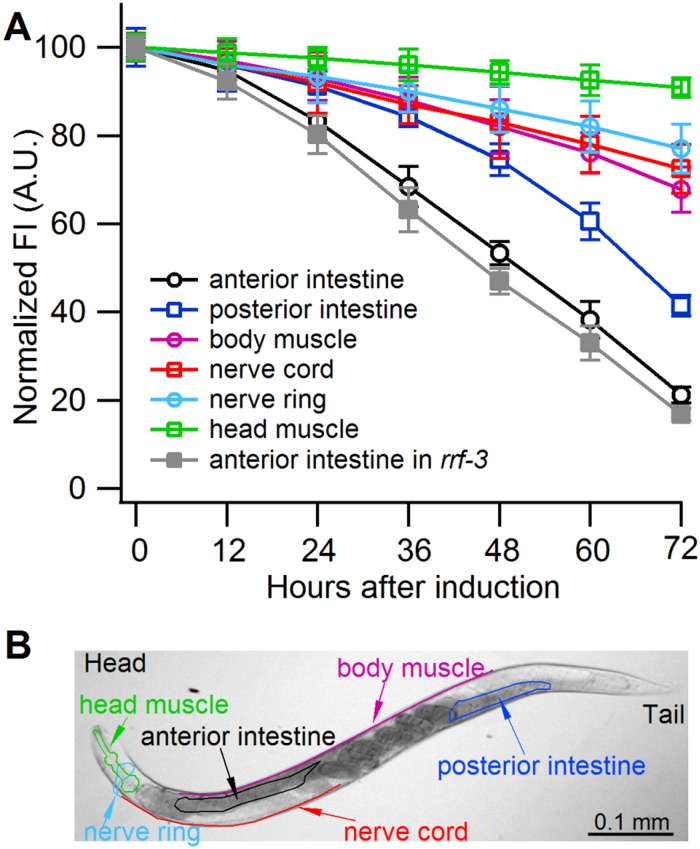
Sense-mediated silencing of *gfp* expression in transgenic *C*. *elegans*. (**A**) Normalized GFP fluorescence intensity (FI) over time after feeding four different *gfp*-transgenic strains of *C*. *elegans* with *E*. *coli* expressing *gfp* mRNA. GFP fluorescence intensity was set at 100% at the beginning of each experiment (0 h), and relative changes in fluorescence intensity were followed over time. Each GFP fluorescence curve corresponds to a different tissue type. *gfp* expression was driven by promoters specifically active in (i) anterior and posterior intestine (*C*. *elegans* strain RT476), (ii) body muscle (strain SJ4157), (iii) nerve cord and ring (strain NM2415), or (iv) head muscle (strain NP738). Introgression of a deletion in *rrf-3*, a gene encoding a cellular RNA—dependent RNA polymerase homolog that interferes with RNAi in *C*. *elegans* [74, 75], to the transgenic strain RT476 resulted in no further enhancement of *gfp* silencing. At least 25 individual nematodes were assessed from each transgenic line at each time point in three independent experiments. Bars indicate S.E.M. (**B**) Morphology of examined body parts in *C*. *elegans*.

Owing to systemic spread of silencing in *C*. *elegans*, large numbers of the nematodes actively performing RNAi can be obtained by feeding them bacteria engineered to express high levels of a specific dsRNA [6]. Because CSR3 suppressed only sense-mediated RNAi in *N*. *benthamiana*, however, an *E*. *coli* strain engineered to express high levels of *gfp* mRNA was used as an RNAi inducer in *C*. *elegans*. These bacteria were fed to four transgenic strains of *C*. *elegans* expressing *gfp* under different tissue-specific promoters (Fig. 3A, S1 Table). *gfp* silencing was observed in all four strains, whereas no detectable reduction of GFP fluorescence was observed in nematodes fed bacteria transformed with an empty (no insert) plasmid or with a promoterless plasmid as tested with strain RT476 (S1A,B Fig.). The greatest (*ca*. 5-fold) reduction of GFP fluorescence was observed in strain RT476 (Fig. 3A, S1B Fig.) expressing *gfp* under the intestine-specific promoter *vha-6* [27]. Strand-specific reverse transcription—PCR (RT-PCR) detected exclusively *gfp* sense transcripts (mRNA) in the *gfp*-transformed bacteria, and no antisense *gfp* transcripts were detected (S1C Fig.), suggesting that efficient sense-mediated silencing of *gfp* could be achieved in intestine tissue of *C*. *elegans*. Hence, strain RT476 was chosen for use in the RNAi suppression experiments.

**Fig 4:**
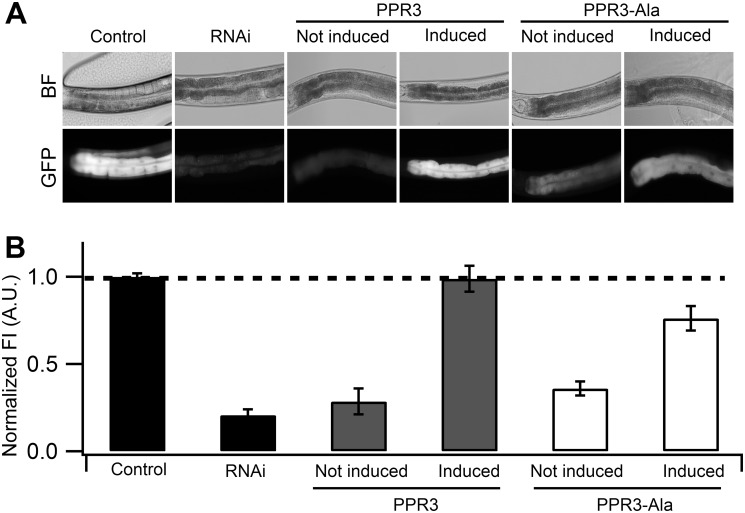
Suppression of RNAi in *C*. *elegans* by PPR3 and PPR3-Ala. The *gfp*-transgenic strain RT476 was transformed to express PPR3 or PPR3-Ala under a heat shock—inducible promoter. Sense-mediated silencing of *gfp* expression was induced in the nematodes by feeding them *E*. *coli* expressing *gfp* mRNA (RNAi). In Control, *gfp*-transgenic nematodes were fed bacteria harboring an insert-less plasmid. After 24 h, expression of PPR3 and PPR3-Ala was induced by heat shock in the *gfp*-silenced nematodes, which recovered GFP fluorescence by 72 h post-induction. (**A**) *gfp*-transgenic *C*. *elegans* (strain RT476) in bright field (BF) to observe morphology or under UV illumination to observe GFP fluorescence. The anterior intestine and posterior intestine are oriented to the left and right, respectively (see also Fig. 3B). (**B**) Normalized average GFP fluorescence intensity (FI) shown using arbitrary units (A.U.) in four independent experiments. Two transgenic lines expressing each protein were included in the experiments. Error bars indicate S.E. (n = 38–50). GFP fluorescence intensity is significantly higher in the nematodes induced to express PPR3 than PPR3-Ala, but in both it is higher than in uninduced controls (Student’s t-test, p < 0.001). Dashed line indicates the level of GFP fluorescence in control.

Strain RT476 was stably transformed with a gene encoding PPR3, PPR3-Ala, CSR3, or CSR3-Ala placed under the heat shock—inducible promoter *mtl-2* [28]. Two independent transgenic lines of the same strain expressing each protein were used for the experiments. Silencing of *gfp* was induced by feeding the nematodes with bacteria expressing *gfp* mRNA, and 72 h later expression of the viral protein was induced by heat shock. GFP fluorescence was observed 24 h after inducing viral protein expression. In four independent experiments, PPR3 restored GFP expression, as indicated by GFP fluorescence intensity that was similar to the *gfp*-transgenic strain RT476 fed bacteria containing a plasmid lacking an insert (control; Fig. 4). PPR3-Ala partially restored GFP expression, with GFP fluorescence intensity attaining ~75% of the level measured in the control. In contrast, no enhancement of GFP fluorescence was observed in the *gfp*-silenced nematodes that were transformed with PPR3 or PPR3-Ala but not induced to express these proteins (Fig. 4).

**Fig 5:**
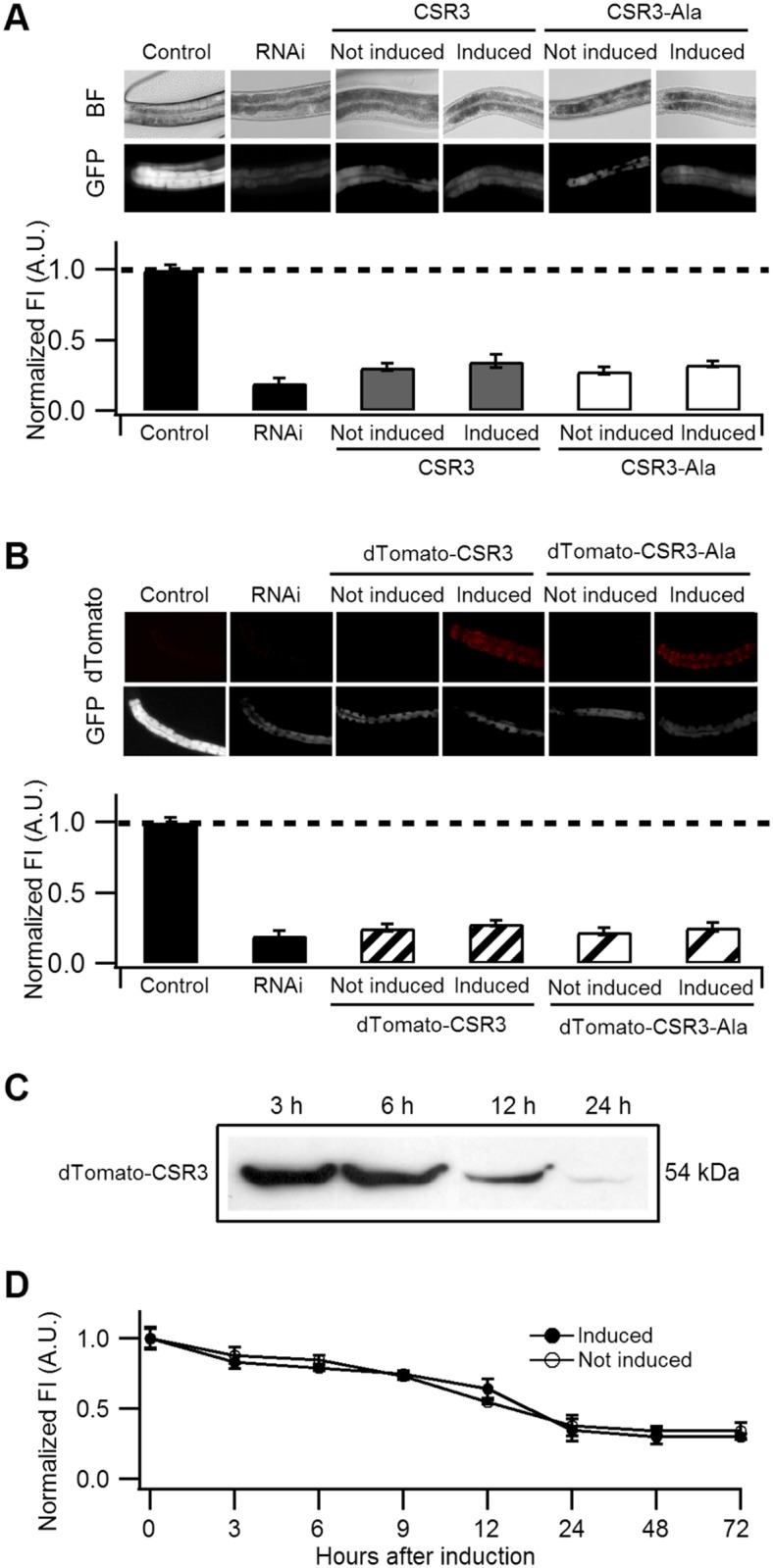
Ability of CSR3 and CSR3-Ala to interfere with *gfp* silencing in *gfp*-transgenic *C*. *elegans* (strain RT476) expressing GFP under an intestine-specific promoter. (**A, B**) Upper panel: representative images of *C*. *elegans* intestine using bright field (BF) illumination or UV light to observe fluorescence of GFP or the red fluorescent protein dTomato. Lower panel: normalized average intensity of GFP fluorescence in four independent experiments. Error bars indicated S.E. (n = 35–50). In (**A**), the *gfp*-transgenic strain RT476 was transformed to express CSR3 or CSR3-Ala under a heat shock—inducible promoter, and two transgenic lines expressing each protein were included in experiments. Sense-mediated silencing of *gfp* expression was induced in the nematodes by feeding them *E*. *coli* expressing *gfp* mRNA (RNAi), which reduced GFP fluorescence significantly (*gfp*-transgenic nematodes fed bacteria harboring an insert-less plasmid were used as a control) (Student’s t-test, p < 0.001). At 72 h, expression of CSR3 and CSR3-Ala was induced in the *gfp*-silenced nematodes by heat shock. At 24 h post-induction, however, no significant difference (Student’s t-test) in GFP fluorescence was detected in nematodes expressing CSR3 or CSR3-Ala as compared with the *gfp*-silenced strain (RNAi). In (**B**), similar experiments as above were carried out with the *gfp*-transgenic strain RT476 transformed to express CSR3 or CSR3-Ala fused with dTomato. No recovery of GFP fluorescence was observed. (**C**) Immunoblot analysis using a rabbit polyclonal antibody specific to CSR3 detected the dTomato-CSR3 fusion protein in nematodes (B) in which dTomato-CSR3 expression was induced. (**D**) Simultaneous induction of CSR3 expression and GFP-silencing. CSR3 was not able to delay silencing.

Expression of CSR3 or CSR3-Ala did not result in recovery of GFP fluorescence in *gfp*-silenced *C*. *elegans*, as observed 24 h post-induction of the viral protein expression (Fig. 5A). When CSR3 and CSR3-Ala were expressed as a fusion product with the red fluorescent protein dTomato [29] in *C*. *elegans* strain RT476, the readily detectable red fluorescence occurring 24 h after induction indicated high levels of protein expression. As observed before, GFP fluorescence did not recover in the four independent experiments (Fig. 5B). Immunoblotting revealed high amounts of dTomato-CSR3 in the nematodes by 3 h post-induction followed by a gradual decline (Fig. 5C), which is consistent with previous studies carried out using the same promoter [30, 31]. Taken together, these results suggested that CSR3 was unable to reverse sense-mediated silencing in *C*. *elegans*.

CSR3 was tested for interference with induction of sense-mediated RNAi by expressing CSR3 simultaneously with induction of *gfp* silencing in *C*. *elegans*. In two independent experiments, GFP fluorescence declined similarly with time (0 to 72 h) irrespective of whether the nematodes were induced to express CSR3 (Fig. 5D), providing no evidence of obstruction with RNAi.

**Fig 6:**
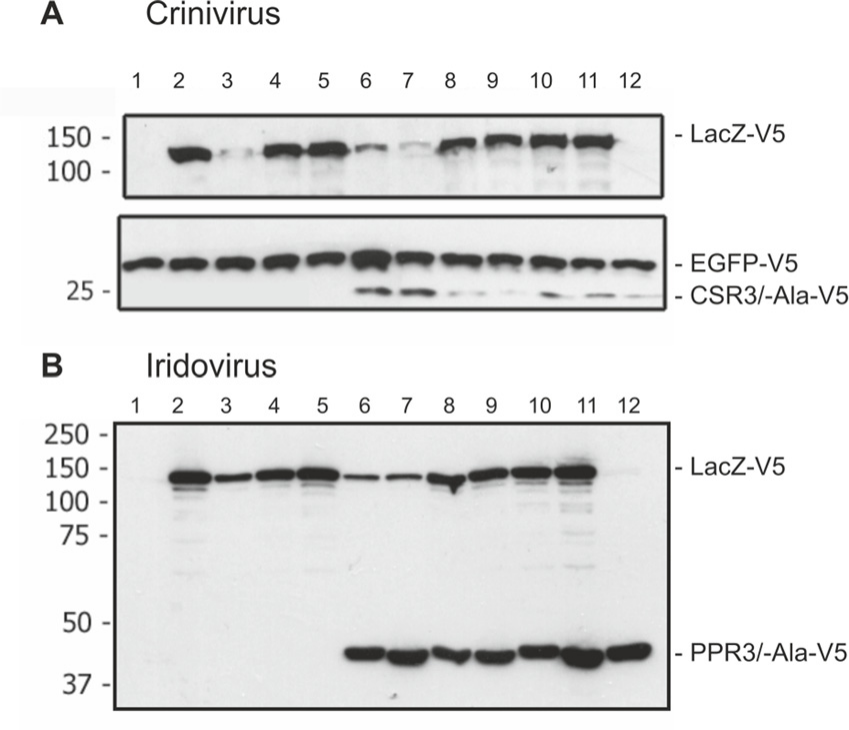
Immunoblot analysis of proteins in *Drosophila melanogaster* S2 cells transfected to co-express LacZ and viral RNase III proteins. S2 cells were transfected to co-express sense and antisense transcripts of LacZ to silence LacZ expression, and they were additionally co-transfected to express (**A**) CSR3 or CSR3-Ala (Crinivirus) or (**B**) PPR3 or PPR3-Ala (Iridovirus) and tested for their ability to suppress *LacZ* silencing. In some experiments, GFP was co-expressed as an internal control to verify similar levels of protein expression, as shown in A. All genes were expressed under a metallothionein promoter, which allowed simultaneous induction of expression of all genes by treatment of cultures with CuSO_4_. Cells were harvested for analysis 72 h post-induction. All proteins were expressed with the V5 tag fused to the C-terminus and detected by immunoblotting using monoclonal anti-V5. Co-expression of LacZ from two plasmids in an attempt to induce sense-mediated silencing (lane 4, both panels) or co-expression of LacZ and antisense luciferase (*luc*) transcripts (lane 5 in both panels) had no significant effect on LacZ expression level. In contrast, LacZ expression was reduced significantly in the cells co-transfected with LacZ and the anti-LacZ construct (lanes 3, 6 and 7), irrespective of whether CSR3 or CSR3-Ala (lanes 6 and 7 in panel A) or PPR3 or PPR3-Ala (lanes 6 and 7 in panel B) were co-expressed in the same cells. These results indicated that the tested viral proteins could not suppress antisense-mediated gene silencing in *D*. *melanogaster*. S2 cells were transfected or co-transfected for expression of the following constructs. “RNase III” refers to CSR3 (panel A) and PPR3 (panel B): lane 1, an empty plasmid; lane 2, LacZ; lane 3, LacZ and antisense-LacZ; lane 4, LacZ and LacZ; lane 5, LacZ and antisense-luc; lane 6, LacZ and antisense-LacZ and RNase III; lane 7, LacZ and antisense-LacZ and RNase III-Ala; lane 8, LacZ and LacZ and RNase III; lane 9, LacZ and LacZ and RNase III-Ala; lane 10, LacZ and RNase III; lane 11, LacZ and RNase III-Ala; lane 12, RNase III. Size markers (kDa) are shown to the left of each blot.

We tested CSR3 and PPR3 also for their ability to interfere with antisense-mediated RNAi of *LacZ* in the S2 cells of *D*. *melanogaster*. The *LacZ* gene was co-expressed in sense and antisense orientations from two plasmids, which induced antisense-mediated silencing against the gene. Analysis of proteins extracted from the cell cultures 72 h post-induction showed that antisense-mediated silencing significantly reduced *LacZ* expression, as compared with controls including co-expression of *LacZ* and the luciferase gene (*luc*), or co-expression of sense *LacZ* transcripts from two plasmids (Fig. 6). However, transfection of S2 cells with an additional plasmid expressing CSR3, PPR3, or the mutant CSR3-Ala or PPR3-Ala did not interfere with *LacZ* silencing and enhance *LacZ* expression in *D*. *melanogaster* (Fig. 6). These results indicated that CSR3 and PPR3 were unable to suppress antisense-mediated RNAi in *D*. *melanogaster*.

### PPR3 Suppresses Antiviral RNAi

**Fig 7:**
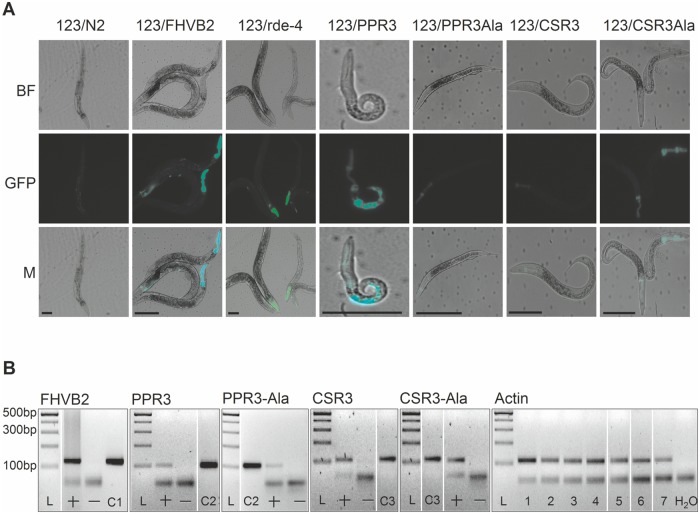
Suppression of antiviral RNAi by PPR3 in *C*. *elegans*. (**A**) GFP fluorescence and stunted growth of the heat shock—treated *C*. *elegans* strain 123/PPR3 expressing PPR3 and a mutated flock house virus (FHV) genome (FR1gfp), in which the *B2* gene for an RNAi suppressor had been replaced with *GFP*, and in *C*. *elegans* strain 123/FHVB2 expressing FHV *B2* in the FR1gfp background under control of a heat shock—inducible promoter. Strain 123/rde-4 is defective in RdRp function, hence deficient in RNAi and allows GFP expression in pharyngeal cells. In contrast, the lines expressing PPR3-Ala, CSR3, or CSR3-Ala show only faint signals of the pharyngeal-specifically expressed cyan fluorescent protein used as a co-injection marker. Images taken under a microscope in bright field (BF) or with a GFP fluorescence filter (GFP) were merged (M). Scale bars, 100 μm. (**B**) Transgene expression was detected by RT-PCR on DNase-treated total RNA extracted from worms. PCR template (100 ng) was cDNA (indicated by a +) or extracted RNA that was DNase-treated but not reverse transcribed (—). Positive controls (DNA): C1, FHV RNA1 plasmid (includes *B2*); C2, PPR3 plasmid; C3, CSR3 plasmid. Detection of actin mRNA was used as an additional control. PCR products were verified by sequencing. L, Gene Ruler DNA ladder mix.

The transgenic *C*. *elegans* strain 123 contains a heat shock-inducible Flock House virus (FHV, family *Nodaviridae*) RNA1 replicon termed FR1gfp [32, 33]. In FR1gfp, the coding sequence of FHV B2, an RNAi suppressor, is replaced with *GFP* coding sequence, making the virus unprotected against RNAi [32]. However, in the presence of an RNAi suppressor, the replication of FR1gfp is restored to produce green fluorescence, making 123 an ideal strain to identify viral RNAi suppressors in *C*. *elegans* [32, 33]. The *C*. *elegans* strain 123/FHVB2 contains both the FR1gfp replicon transgene and a heat inducible FHV B2 transgene which is able to rescue FR1gfp replication and thus GFP expression in pharynx and muscle cells 24 h post heat shock (Fig. 7A, positive control) [33]. The *C*. *elegans* strain 123/rde-4 contains a null allele of *rde-4*. Because *rde-4* encodes a dsRNA-binding protein that plays an essential role in RNAi, FR1gfp replication and GFP production in 123/rde-4 are restored (Fig. 7A, positive control) [32]. We transformed worms of strain 123 with the genes for CSR3, CSR3-Ala, PPR3 and PPR3-Ala placed under the heat-shock—inducible promoter *mtl-2* and included two independent progeny lines of each in further experiments (lines 123/CSR3, 123/CSR3Ala, 123/PPR3 and 123/PPR3Ala, respectively). Strong GFP fluorescence was observed in the intestine of the progeny of line 123/PPR3 at 24 h post-heat shock, indicating suppression of RNAi-based antiviral defense (Fig. 7A). The heat shock-treated worms of line 123/PPR3 had stunted growth and fewer progeny were obtained than for other transgenic lines, suggesting harmful physiological effects of PPR3. GFP expression also was detected in the progeny of lines 123/FHVB2 and 123/red-4, as expected (Fig. 7A). The remaining lines displayed no GFP fluorescence, but faint signals of the pharyngeal cyan fluorescent protein used as a co-injection marker (*e*.*g*., line 123/CSR3Ala in Fig. 7A). The heat-induced expression of transgenes was verified by RT-PCR in all transgenic lines (Fig. 7B).

CSR3 suppresses antiviral RNAi efficiently in plants [24], but whether PPR3 is able to do the same was tested in this study. Tobacco rattle tobravirus (TRV) has a bipartite (+)ssRNA genome and infects a wide range of plant species. TRV RNA1 contains coding sequences for a replicase and two RNAi suppressors, namely 16K, the main suppressor of RNAi [34–36], and 29K that also acts as a viral cell-to-cell and long distance movement protein [37]. TRV RNA1 can infect plants systemically without RNA2, but RNA2 enhances accumulation of RNA1 in plant tissues by an unknown mechanism [37]. TRV-M1 is a TRV RNA1 replicon lacking the coding sequence of 16K and is only weakly protected against RNAi [37]. One half in the full-grown leaves of *N*. *benthamiana* was agroinfiltrated for co-expression of TRV-M1 and PPR3, or TRV-M1, TRV RNA2 and PPR3. The other half of the leaf was agroinfiltrated with controls, namely TRV-M1 only, or with TRV-M1 and TRV RNA2, respectively. Samples of infiltrated leaf tissue had to be collected at 3 d.p.i., because longer co-expression of TRV-M1 and PPR3 induced extensive necrosis of the leaf tissue. The concentrations of TRV RNA1 were estimated by quantitative reverse transcription PCR, which showed that TRV-M1 amounts were enhanced in the presence of PPR3, as compared with the controls (S2 Fig.). Although the necrotic symptoms enforced an early termination of the trials and consequently the differences were not statistically significant, there was a clear and consistent tendency in all three experiments of enhanced TRV RNA1 accumulation in the presence of PPR3.

## Discussion

The cellular RNaseIII—like endoribonucleases belonging to Class 3 (called Dicers) play a key role in RNAi, which functions as a non-virus-specific, basal antiviral defense mechanism in plants [1] and invertebrates such as *C*. *elegans* [2, 3, 38] or insects including *D*. *melanogaster* [39, 40]. Recent studies suggest also an antiviral role of RNAi in vertebrates [41, 42]. Dicers recognize long dsRNA molecules, including those of viral origin, and cleave them to yield 21- to 25-nt siRNAs pivotal in targeting RNAi to silence the homologous viral genomes. PPIV and SPCSV are unrelated viruses differing in genome structure and gene content. PPIV belongs to iridoviruses (family *Iridoviridae*) containing a dsDNA genome that typically codes for *ca*. 100 proteins [19]. SPCSV, in turn, has a bipartite (+)ssRNA genome encoding up to 12 proteins [43]. However, common to PPIV and SPCSV are the Class 1 RNaseIII homologs (PPR3 and CSR3, respectively) produced by both viruses. dsRNA-degrading activity develops in the host cells at an early stage of infection with *Frog virus 3*, the type species of genus *Ranavirus* (*Iridoviridae*) encoding a homolog of RNaseIII [19], and the RNaseIII activity of a homologous protein of rock bream iridovirus was recently reported [20]. In SPCSV, the subgenomic RNA coding for CSR3 is expressed early at infection of plants [43]. The early activation of RNaseIII expression is consistent with its role in interferences with antiviral RNAi, which is supported by our results. They show that PPR3 suppresses antiviral RNAi in *C*. *elegans* and *N*. *benthamiana* and hence enhances accumulation of the RNAi suppression deficient FHV and TRV replicons, respectively. Furthermore, our previous studies have shown that RNAi is suppressed and antiviral defense against many unrelated viruses is eliminated in virus-resistant sweet potato plants transformed to express CSR3 [24]. Finally, it is remarkable that PPR3 (this study) and CSR3 [24] suppress antiviral RNAi autonomously, in the absence of PPIV or SPCSV infection, respectively, which excludes a role for other viral proteins, or perturbation of cellular homeostasis caused by virus infection, in the observed interference with RNAi.

PPIV and SPCSV have unrelated host ranges including poikilothermic animals and plants, respectively. Class 1 RNaseIII—like proteins exhibiting endoribonuclease activity on dsRNA have been reported in *Paramecium bursaria chlorella virus* that infects algae [44], *Diadromus pulchellus ascovirus* [45] and *Heliothis virescens ascovirus* (HvAV-3e) [22] that are DNA viruses infecting insects, and rock bream iridovirus that infects fish [20]. Involvement of the HvAV-3e RNaseIII in suppressing RNAi has been suggested based on results showing that the dsRNA-induced silencing of *gfp* was suppressed in an insect cell line (*Spodoptera frugiperda* Sf9) infected with a baculovirus engineered to express HvAV-3e RNaseIII [22]. Also, many other viruses in the family *Iridoviridae* that infect poikilothermic animals, insects, or other invertebrates [19] and the viruses of family *Ascoviridae* that infect larvae of lepidopteran insects [46] have genes predicted to encode Class 1 RNaseIII—like proteins containing the conserved motifs of Class 1 RNaseIIIs, but the overall amino acid sequence similarity is low [22]. Taken together, interference with RNAi has now been demonstrated with RNaseIIIs of SPCSV, PPIV and HvAV-3e representing unrelated taxa of RNA and DNA viruses. These results suggest that, in general, viral RNaseIII endoribonucleases function as suppressors of RNAi. They represent the first group of RNAi suppressors that are homologous in unrelated viruses.

Mutations in the highly conserved RNase signature motif [22] in CSR3-Ala and PPR3-Ala prevented cleavage of ds-siRNA by both enzymes, but PPR3-Ala could still cleave long dsRNA substrates and partially suppress sense-mediated silencing in *N*. *benthamiana* and reverse silencing in *C*. *elegans*. PPR3 and CSR3 differ greatly for the size (371 and 229 residues, respectively) and amino acid sequence, but share conserved domains characteristic of Class 1 RNaseIII enzymes. Whereas CSR3—with a single dsRNA-binding domain—resembles the Class 1 RNaseIII of *E*. *coli* [21, 22], PPR3 contains two putative dsRNA-binding domains and resembles AtRTL2 of *Arabidopsis thaliana* [47]. On the other hand, little sequence variability is observed among the RNaseIII proteins of the different iridoviruses or between the isolates of SPCSV. The RNaseIII proteins of PPIV (NCBI sequence database accession KC191670), *Frog virus 3* (AY548484), Rana grylio iridovirus (JQ654586), Soft-shelled turtle iridovirus (EU627010.1), Andrias davidianus ranavirus isolate 2010SX (KF033124) and Chinese giant salamander iridovirus (KF512820) vary only at three amino acid positions that are not situated in the RNA-binding and catalytic domains. The deduced CSR3 amino acid sequences characterized in 69 isolates of SPCSV were 98.7–100% identical and no sequence variability was observed in the RNA-binding and catalytic domains [48]. Therefore, PPR3 and CSR3 used in this study stood for the typical sequences of the respective types of RNaseIII enzymes. The difference in RNAi suppression, however, may be explained by the two dsRNA-binding domains of PPR3 as compared with a single dsRNA-binding domain in CSR3. Two dsRNA-binding domains may allow more efficient sequestering of ds-siRNA, and/or competition with Dicer/AGO for binding long dsRNA, which could be significant because siRNA binding is a common mechanism by which viral proteins suppress RNAi [17, 49]. Despite these differences, results with PPR3 and CSR3 were consistent in that the similar mutations introduced to the RNase signature motif abolished cleavage of ds-siRNA and debilitated RNAi suppression, suggesting that cleavage of ds-siRNA is required for efficient suppression of RNAi. Cleavage products of Class 1 RNaseIII are shorter (~15 bp) than those generated by Dicers and are not functional in RNAi [50, 51]. The suggested RNAi suppression activity of the HvAV-3e RNaseIII may function similarly because it cleaves 21-nt ds-siRNA [22].

Our results suggest that PPR3 is more versatile than CSR3 for suppressing RNAi. Whereas PPR3 and CSR3 both suppressed sense-mediated RNAi in plant tissue, PPR3 also suppressed dsRNA-mediated silencing. Furthermore, in contrast to CSR3, PPR3 was able to reverse silencing in plant tissue and *C*. *elegans*. These results suggest a different level of host specificity in the action of CSR3 and PPR3. However, both PPR3 and CSR3 failed to suppress RNAi in *D*. *melanogaster*. Previous studies have shown that the non-homologous proteins P15, P19, and P21 encoded by unrelated plant RNA viruses, the B2 protein of FHV (insect virus), and the 1A protein of *Drosophila C virus* suppress dsRNA-mediated RNAi in *D*. *melanogaster* [52]. All these plant viral proteins may suppress RNAi by binding and sequestering virus-derived siRNAs, which has been particularly well characterized for P19 [53]. Additionally, B2 binds dsRNA, which 1A may bind preferably [52]. These results are consistent with the conclusion that RNAi suppression exhibited by the RNaseIII enzymes was connected to their catalytic activity on dsRNA and ds-siRNA rather than RNA binding, which is not efficient enough to suppress RNAi. Furthermore, while the RNAi pathways in plants, *C*. *elegans*, and *D*. *melanogaster* conferring resistance to viruses [1–3, 38, 40, 54] include conserved components, there are also differences. For example, the RdRp mediated amplification of RNAi involves distinct mechanisms in plants and animals [12, 15, 55]. Non-optimal pH [56] and possible incompatible interactions between the viral silencing suppressors and cellular proteins [49] may affect catalytic activity of CSR3 and PPR3 and explain the differences in suppression of RNAi in a non-host cellular environment. Incompatible host interactions may also cause the necrotic response observed in the leaves co-expressing PPR3 and the TRV replicon.

Taken together, our results suggest a hypothesis in which the viral Class 1 RNaseIII may compete with Dicer for processing the long virus-derived dsRNA and AGO for binding ds-siRNAs, but especially destroys the ds-siRNA to prevent loading of RNA-induced silencing complexes (RISC), production of secondary siRNAs and amplification of RNAi [4, 12, 16, 32, 57]. The versatile functions of PPR3 in plants and animals suggest that it is less dependent on host factors than CSR3. Furthermore, the results of our study show that the viral Class 1 RNaseIIIs are unique among viral RNAi suppressors because they are homologous in unrelated RNA and DNA viruses and can be detected in viral genomes using gene modeling and protein structure prediction programs. Our results underscore the importance of their catalytic activities in suppressing RNAi. Understanding of this novel mechanism of RNAi suppression may inform means of controlling the diseases and economic losses which the RNaseIII-containing viruses cause in animal and plant production [58–60].

## Materials and Methods

### Cloning the Viral RNaseIII Genes for Expression in Plants

The binary plasmids containing the RNaseIII gene (*CSR3*) of SPCSV and the corresponding mutated gene (*CSR3-Ala*) have been described [21, 24]. In the binary vector (pKOH200), the coding region is flanked by the *Cauliflower mosaic virus* 35S promoter and the 3′ terminator region (3’ *nos*) of the nopaline synthase gene.

PPIV-infected tissue was obtained from newly hatched pike-perch (*Stizostedion lucioperca*) provided by H. Tapiovaara, Finnish Food Safety Institute Evira, Finland. Multiplication and isolation of the virus were carried out in a blue fry gill (BF-2) cell line [61]. Cell culture supernatants were collected and the DNA purified using the QIAamp DNA Mini kit (Qiagen). The RNaseIII ORF (*PPR3*) was PCR-amplified from PPIV using primers PPR3 *Not*I fwd and PPR3 *Fse*I +StrepII rev (S1 Table) designed based on the flanking conserved genome regions identified by comparison with the genome sequences of Frog virus 3 (FV-3; GenBank AY548484) [62], Tiger frog virus (AF389451), and *Ambystoma tigrinum* virus (NC_005832) [63]. The amplified PCR product was treated with *Not*I and *Fse*I, which allowed cloning of *PPR3* in an intermediate *E*. *coli* plasmid (pKOH122) containing the 35S promoter and 3′*nos* sequences [64]. The gene was sequenced (GenBank as accession no. KC191670). *PPR3* was mutated using the QuickChange Site-Directed Mutagenesis System (Stratagene) with primers PPR3-Ala fwd and rev (S1 Table) to introduce two amino acid substitutions (E44A and D51A) into the conserved catalytic site and express the mutant PPR3-Ala analogous to CSR3-Ala [21].

The cassettes ‘35S-PPR3-NosT’ and ‘35S-PPR3-Ala-NosT’ were excised from pKOH122 and subcloned into the binary vector pKOH200 for plant transient expression [64], resulting in plasmids pKOH200 PPR3 and pKOH200 PPR3-Ala. For immunodetection, a StrepII-tag (amino acid sequence WSHPQFEK) was fused with the C-terminal end of both PPR3 and PPR3-Ala.

### Constructs for Recombinant Protein Expression in *E*. *coli*

For RNaseIII expression in *E*. *coli*, the full-length ORFs were amplified from the respective pKOH200 vectors (see above) and cloned fused with a C-terminal His-tag in pET11d+ vector as described [21, 24]. Amplification and cloning of the genes *PPR3* and *PPR3-Ala* followed a similar procedure except an N-terminal His-tag fusion was used. The vectors (pET11d^+^ His-PPR3, pET11d^+^ His-PPR3-Ala, pET11d^+^ CSR3-His, pET11d^+^ CSR3-Ala-His) were transformed into *E*. *coli* strain BL21 (DE3 PLUS RIL; Qiagen) and expressed (for details, see below).

### Constructs for Transformation of *C*. *elegans*

The coding regions for PPR3 and PPR3-Ala were amplified from pET11d^+^ His-PPR3 and pET11d^+^ His-PPR3-Ala using primers 54_01 PPR3 *Xba*I fwd and 54_01 PPR3 *Nhe*I rev (S1 Table). The PCR products were digested with *Xba*I and *Nhe*I and introduced in the corresponding sites of vector pPD54_01 to generate pPD54_01 PPR3 and pPD54_01 PPR3-Ala, respectively.

The full-length *CSR3* and the *CSR3-Ala* coding regions were amplified from pET11d^+^ CSR3-His and pET11d^+^ CSR3-Ala-His, respectively, using primers 54_01 CSR3 *Xba*I fwd and 54_01 CSR3 *Xma*I rev (S1 Table). The constructs were cloned downstream of the *mtl-2* promoter of the pPD54_01 expression vector (Plasmid 1507; Addgene, Cambridge, MA) using the *Xba*I and *Xma*I sites, resulting in plasmids pPD54_01 CSR3 and pPD54_01 CSR3-Ala. In addition, CSR3 and CSR3-Ala fusion proteins were generated using the dTomato tag [29]. Briefly, amplification of the *dTomato* coding sequence from plasmid pRSET dTomato was carried out with the primer pair 54_01 dTomato-CSR3 *Xba*I fwd and 54_01 dTomato-CSR3 *Xba*I rev (S1 Table). The PCR products were digested with *Xba*I and ligated in the corresponding sites of pPD54_01 CSR3 and pPD54_01 CSR3-Ala. All constructs were verified by sequencing.

The promoterless construct pET24b^+^ GFPopt—ΔT7 was obtained by digesting pET24b^+^ GFPopt with *EcoR*I and *SgrA*I. The insert was omitted, and the plasmid was blunt-ended with Klenow polymerase (NEB) and religated using T4 ligase (NEB). This promoterless plasmid was used as a negative control for gene expression in the transformation of *C*. *elegans*. All constructs and mutations were verified by sequencing (Haartman Institute, DNA sequencing unit, University of Helsinki).

### Strains of *C*. *elegans* and Germline Transformation

Strains of *C*. *elegans* were maintained as described [65]. For analyzing the silencing effects in different tissues, four different strains were employed (S1 Table). For silencing suppression experiments, the *C*. *elegans* strain RT476 Is[Pvha-6:gfp::RAB-7] was used for stable transformation. In this strain, *gfp* was constitutively expressed under the intestine-specific promoter *vha-6* and marked the RAB-7-positive endosomes in *C*. *elegans* intestinal cells. Vector pPD54_01 (Addgene) was employed for the stable germline transformation allowing heat shock—inducible expression of the protein in intestine. The constructs pPD54_01 CSR3, pPD54_01 CSR3-Ala, pPD54_01 PPR3, and pPD54_01 PPR3-Ala were stably introduced to the nematodes through germline transformation using the standard microinjection method [66] delivering 50 ng/μl of the construct of interest. In all experiments, 50 ng/μl pRF4 plasmid was co-injected. pRF4 harbors the dominant *rol-6* (*su1006*) allele that causes a readily distinguishable roller phenotype in transgenic animals and serves as a co-transformation marker [67]. The transgenic strains FR1gfp (denoted as 123/N2), 123/FHVB2 and 123/rde-4 of *C*. *elegans* have been described [32, 33]. 123/N2 animals were injected with pPD54_01 CSR3, pPD54_01 CSR3-Ala, pPD54_01 PPR3 or pPD54_01 PPR3-Ala (100 ng/μl). The lines are denoted as 123/CSR3, 123/CSR3Ala, 123/PPR3 and 123/PPR3Ala, respectively. The plasmid encoding Pmyo-2::cfp pharyngeal CFP [68] was co-injected as an injection marker (50 ng/μl). Transgenes were maintained as extra-chromosomal arrays, and two independent lines carrying each transgene were isolated and analyzed.

### RNAi and RNAi Suppression Assay in *C*. *elegans*

*E*. *coli* strain HT115 was transformed with pET24b^+^GFPopt or with (i) the pET24b^+^ empty vector (Novagen) or (ii) pET24b^+^ GFPopt—ΔT7 as controls. In pET24b^+^ GFPopt, *gfp* was inserted between the T7 promoter and terminator sequences. Transcription was induced in HT115 by growing the bacteria on nematode growth medium plates containing 1 mM IPTG and appropriate antibiotics (as detailed below) leading to the production of sense-*gfp* transcripts.

RNAi was initiated by feeding nematodes with sense-*gfp*-expressing bacteria. For RNAi suppression experiments, *Is[P*_*vha-6*_:*gfp*::*RAB-7]* (S1 Table) and RNaseIII transgenic animals were used. To synchronize the stage of the examined individuals, 4–6 adult nematodes were transferred to each feeding plate for 6 h. The hatched eggs grew to adults within 3 days, and heat shock was carried out by incubating the nematodes 2–3 h at 33–34°C (BioRAD Mini Incubator). GFP fluorescence was recorded at different times after heat shock induction, depending on the experiment. All lines of the transgenic nematode strains were treated and tested in duplicate in each assay in at least three independent experiments.

To test suppression of RNAi, expression of PPR3, PPR3-Ala, CSR3, CSR3-Ala and FHV B2 was induced by heat shock of worms at the temperature mentioned above, as described [32]. The experiment was carried out twice.

### Imaging and Microscopy of *C*. *elegans*

Static microscopic images were acquired using an OLYMPUS AX70 microscope with a 20× objective. The fluorescence signal was quantified and analyzed using ImageJ software (NIH). The total pixel intensity in wild-type worms was set to an arbitrary fluorescence unit of 1.0 to enable comparison with other strains. Representative images of the *C*. *elegans* intestine were taken with 488 nm excitation (emission 520 nm) for quantification of GFP fluorescence at different time points and with 568 nm excitation (emission 595 nm) for visualization of dTomato fluorescence. Imaging data analysis was conducted using IGOR Pro (Wavemetrics) or EXCEL (Microsoft) software. The mean value ± S.E.M. of the indicated experiments was calculated. Statistical significance was evaluated using the Student’s t-test.

In the study of suppression of RNAi, worm progeny were imaged 24 h post-heat shock with a Zeiss Axioplan 2 microscope using a 10× 0.3 NA objective.

### Verification of Sense *gfp* Transcripts Expressed by Bacteria and *C*. *elegans*

*E*. *coli* strain HT115 harboring empty pET24b^+^ or pET24b^+^ GFPopt was grown in liquid Luria Bertani medium containing 50 mg/l tetracycline and 50 mg/l kanamycin overnight at 37°C. Thereafter, 200 μl of the culture was used to inoculate 20 ml of fresh medium and grown until OD_600_ = 0.6. Transcription under the T7 promoter was then induced by addition of 0.1 mM IPTG for 5 h at 37°C. Bacteria were harvested and RNA extracted using the RNeasy Midi kit (Sigma-Aldrich) following the manufacturer’s instructions for total RNA isolation from Gram-negative bacteria. Initial lysis of cells was achieved in 0.25 M Tris-HCl containing 5 mM EDTA (pH 8) including 1 mg/ml lysozyme (Sigma-Aldrich). After elution from the RNA-binding column, DNA was removed by treatment with RNase-free DNase (Promega). After heat-deactivation of DNase, strand-specific cDNA was produced by employing specific primers able to anneal to *gfp* mRNA either for synthesis of sense (pET24b^+^ *EcoR*I rev primer, S1 Table) or antisense (pET24b^+^ *Xho*I fwd primer, S1 Table) transcripts. RT-PCR was done using M-MLV-H^-^mutant reverse transcriptase (Promega) at 55°C. PCR (38 cycles) using Dynazyme II DNA polymerase (Thermo Fisher) was carried out at 57°C with primers pET24b^+^ *Xho*I fwd and pET24b^+^ *EcoR*I rev to detect sense and antisense strands.

Expression of transgenes was tested in *C*. *elegans* by RT-PCR. A total of 15–30 progeny of the transgenic line were collected following heat shock and stored at -80°C. Total RNA was isolated using a Trizol-based extraction method [69]. cDNA was synthesized using random oligo (dT)_18_ primers (Maxima First Strand cDNA Synthesis kit, Thermo Scientific), and 100 ng was used as template in PCR. Primers rtPPR3fwd (5′-TTGGTTGGGAAACTTGCTCG-3′) and rtPPR3rev (5′-CACTCTTGGGCGTAAACACC-3′) were used to detect the transcripts of *PPR3* and *PPR3-Ala* (amplicon size 101 bp), whereas primers rtCSR3fwd (5′-GAGAATCGTTGGTTGGTTGG-3′) and rtCSR3rev (5′-GGGCAGGTTTCTTAATGTGG-3′) were used to detect the transcripts of *CSR3* and *CSR3-Ala* (amplicon size 107 bp). Expression of the transgene *FHVB2* was tested with primers rtB2fwd (5′-ACAACCACGCCACATAACAC-3′) and rtB2rev (5′-GACCATCATCACCGCACTTC-3′) (amplicon size 127 bp). Actin mRNA was detected as an internal control using primers targeting exon 1 (act-1 fwd. 5′-TCGGTATGGGACAGAAGGAC-3′ and act-1 rev. 5′-CATCCCAGTTGGTGACGATA-3′) (amplicon size 108 bp). PCR products were verified by sequencing.

### Protein Expression in *E*. *coli*

Bacteria carrying the expression plasmid were grown and recombinant proteins expressed as described in The QiaExpressionist (Qiagen). Briefly, protein expression was induced by adding 0.1 mM IPTG in the culture medium growing bacteria overnight at 16°C. Bacterial cells were lysed using lysis buffer (50 mM Na_2_H_2_PO_4_, pH 8.0, 300 mM NaCl, 10 mM imidazole) supplemented with a protease inhibitor cocktail [Roche; 2.5 ml of stock solution (1 tablet/10 ml lysis buffer) in 10 ml extract] and lysozyme (Sigma-Aldrich; 1 ml of 10 mg/ml stock solution in 10 ml buffer) and incubated for 2 h on ice. Cells were additionally disrupted and nucleic acids degraded using sonication (50% duty cycle, 5 × 15 sec; SONIFIER, Branson, Cell Disruptor B15, Hielscher). Recombinant proteins were purified by affinity chromatography with Ni-nitrilotriacetic acid agarose (Ni^2+^-NTA) beads (Qiagen) according to the manufacturer’s protocol and finally loaded onto polypropylene columns (Qiagen). Wash buffer (50 mM Na_2_H_2_PO_4_, pH 8.0, 300 mM NaCl) containing increasing concentrations of imidazole (20–50 mM) was used to obtain pure protein. Bound proteins were eluted with strong elution buffer (50 mM Na_2_H_2_PO_4_, pH 8.0, 300 mM NaCl, 500 mM imidazole). Fractions containing high concentrations of pure protein were collected, 25% (v/v) glycerol was added, and the purified proteins were stored at -20°C for later use. The Bradford colorimetric method (Protein Assay, Dye Reagent concentrate, Bio-Rad) was used for protein quantification. Coomassie Brilliant Blue reagent [10% (v/v) glacial acetic acid, 40% (v/v) methanol, 1% (w/v) Coomassie Brilliant Blue G] was used to visualize proteins in SDS-PAGE gels.

### Protein Extraction and Immunoblotting

The protein extraction method to isolate recombinant proteins from transgenic *C*. *elegans* for detection by western blot analysis was optimized to prevent protein degradation. CSR3 was prone to degradation at low pH for protein extraction from crude extracts of *C*. *elegans*. Protease inhibitors have proved ineffective in preventing degradation, for example with aspartic (acidic) proteases exhibiting pronounced activity at low pH in *C*. *elegans* extracts [70]. However, stable buffering of the extraction environment at pH 11 hindered protein degradation during extraction. The extraction buffer contained 2% SDS and 100 mM N-cyclohexyl-3-aminopropanesulfonic acid (Sigma) adjusted to pH 11 with 2 M NaOH.

After heat shock induction of protein expression, samples of ~1000 nematodes were collected at different time points (0 to 24 h) and proteins extracted using 25 μl of the extraction buffer and incubation at 100°C with occasional vortexing. After 5 min of centrifugation at13,000× *g* at room temperature, the supernatant was subjected to analysis with SDS-PAGE (12% acrylamide; running buffer: 0.025 M Tris-HCl, 0.192 M glycine, 0.1% SDS). Separated proteins were electrotransferred to a PVDF membrane (Amersham Hybond-P, GE healthcare) at 70 V (300 mA) in buffer containing 0.0275 M Tris-HCl, 2.4 M glycine, and 20% (v/v) methanol. Immunodetection was conducted with primary polyclonal anti-CSR3 as described [1]. To eliminate nonspecific cross-reactions of the polyclonal antibodies, 10 ml of 2.5% (w/v) BSA in Tris-buffered saline containing 0.1% (v/v) Tween-20 (which included the primary antibody diluted 1:500) was incubated with 1% (w/v) wild-type nematode extract at 4°C for 1 h with shaking. A horseradish peroxidase—conjugated donkey anti-rabbit IgG (GE Healthcare) secondary antibody was used.

Ectopic expression of proteins in plants was confirmed by crushing leaf material (200 mg) in liquid nitrogen and boiling in the presence of 200 μl 2× protein sample buffer for SDS-PAGE [50 mM Tris-HCl (pH 6.8), 100 mM DTT, 2% SDS, 0.005% bromophenol blue, and 10% (v/v)] glycerol]. Protein extract (20 μl) was subjected to SDS-PAGE (12% acrylamide) and transferred to a Hybond-P membrane by electroblotting. Anti-CSR3 and anti-StrepTagII (Stratagene) were used as primary antibodies, respectively, together with a 1% (w/v) extract of healthy *N*. *benthamiana* leaves to lower nonspecific binding. Secondary ECL anti-rabbit IgG conjugated with horseradish peroxidase was used (Amersham).

Signals were detected via ECL chemiluminescence using the SuperSignal West Pico Chemiluminescent Substrate (Pierce/Thermo Fisher Scientific), and membranes were exposed to film (Kodak).

### *In Vitro* RNA Cleavage Assays

Long dsRNA was produced according to the manual of Replicator RNAi kit (Finnzymes) using T7 GFP fwd and Phi6 GFP rev primers (S2 Table) based on the *gfp* sequence using pKOH GFP as a template for PCR, which produced a dsDNA intermediate. The dsDNA was subsequently transcribed and the complementary strand added by using T7 and Phi-6 polymerases, respectively, in a single reaction.

Short dsRNA processing was screened with the synthetic 24-nt *gfp* ds-siRNA: sense, GAGAGGGUGAAGGUGAUGCUACAC; antisense, GUAGCAUCACCUUCACCCUCUCAG. Equal volumes of sense and antisense RNA strands (Sigma; stock 0.1 μg/ml) were incubated at 85°C for 15 min and subsequently cooled to room temperature. The resultant double-stranded oligos contained typical 3′overhangs, 5′OH, and no further modifications.

The cleavage assays were done at least three times with PPR3, PPR3-Ala, CSR3, and CSR3-Ala using optimized endoribonucleolytic cleavage reactions at 37°C, as described [56]. Samples were analyzed by agarose gel electrophoresis (1% for long dsRNA; 4% for short dsRNA), stained with ethidium bromide and visualized by a UV photoimager (Molecular Imager, Gel Doc XR+, Bio-Rad).

### Agroinfiltration

The transgenic *N*. *benthamiana* (line 16c) plants expressing GFP [54] were grown in growth chambers (temperature 18–22°C, 70% relative humidity) with a 16-h photoperiod using sodium high-pressure lamp illumination. The binary vectors were transformed into competent *A*. *tumefaciens* C58C1 (pGV3850) cells by the freeze and thaw method [71] and agroinfiltration was carried out as previously described [24]. Co-infiltrations [72] were done with a mixture of *A*. *tumefaciens* overnight cultures (1:1 ratio) carrying constructs encoding pBIN35S GFP [73] or pKOH200 hpGFP [21] (OD_600_ = 0.5) and one of the test constructs expressing the wild-type and mutant CSR3 proteins (OD_600_ = 0.5) or PPR3 proteins (OD_600_ = 0.1). *Agrobacterium* cultures for expression of PPR3 had to be diluted more than for expression of other proteins, because PPR3 expression caused necrosis, which was not observed with the PPR3-Ala. Dilution of the *Agrobacterium* culture to OD_600_ = 0.1 (rather than only OD_600_ = 0.5) delayed development of necrosis until 4 d.p.i. *A*. *tumefaciens* expressing the β-glucuronidase gene (GUS) was included as a negative control, and the viral silencing suppressors HCpro and p22 [21] were included as positive controls. GFP expression was monitored by epi-illumination using a hand-held UV-lamp (B-100 AP; UVP, Upland, CA) up to 8 d.p.i.

Reversion of silencing was tested by inducing *gfp* silencing with pBIN35S GFP infiltration in 16c transgenic plants, and expressing viral proteins subsequently (after 24 h) by agroinfiltration in the same leaf spots. Reversion of silencing was monitored up to 6 d.p.i. Images were acquired with a CANON EOS 40D digital camera with an EFS 17–85 mm objective and processed using CorelDRAW Graphics Suite X3 and Adobe Photoshop software.

### Detection of RNA by Northern Analysis

Total RNA was extracted from leaf tissue using TRIzol LS Reagent (Invitrogen), also allowing the recovery of the siRNA fraction. High- and low-molecular-weight RNA fractions were separated and the quality was determined by electrophoresis. The RNA samples were subjected to northern blot analysis using *gfp*—specific probes labeled with [^32^P]UTP as previously described [24]. Radioactive signals on membranes were detected by exposure to X-ray film.

### Experiments on *Drosophila* S2 Cells

The genes encoding CSR3, CSR3-Ala, PPR3, and PPR3-Ala were amplified from the corresponding binary vectors using Phusion high-fidelity DNA polymerase (Finnzymes) and primers containing appropriate restriction sites for cloning (CSR3 *EcoR*I fwd, CSR3 *Xho*I rev, PPR3 *EcoR*I fwd, and PPR3 *Xho*I rev; S1 Table). The purified PCR products were cloned into the *Drosophila* expression vectors pAc5.1 and pMT (Invitrogen) in-frame with a C-terminal V5 (GKPIPNPLLGLDST) and hexahistidine tags. Similarly, *LacZ* in the antisense or sense orientation and *luc* (encoding luciferase) in the antisense orientation were cloned into the vectors. All clones were verified by sequencing.

Each RNaseIII plasmid was co-transfected with antisense or sense *LacZ* or with antisense *luc* plasmid into *Drosophila* S2 cells at 2 × 10^6^ cells/ml in 12-well plates with a total of 1 μg of the indicated plasmids using FugeneHD transfection reagent (Roche). Additionally, in some experiments, co-transfection of pMT-EGFP-V5—6xHis plasmid (constructed as above) was used to control transfection efficiency. Expression of the metallothionin promoter of the constructs was induced with 0.6 mM CuSO_4_ for 3 days, after which the cells were collected by centrifugation and lysed in lysis buffer [Tris-buffered saline pH 7.5, 1% (w/v) Triton X-100, 20 mM NaF, 1 mM EDTA] supplemented with Complete Proteinase Inhibitor Coctail (Roche). Protein concentration of the lysate was measured with Bradford Protein Assay (Bio-Rad). Approximately 10 μg of total protein per sample was subjected to 10% SDS-PAGE followed by immunoblotting and detection with anti-V5 (Invitrogen).

### Accession Numbers

Sequence data from this article can be found in the GenBank database under the following accession numbers: PPR3 (KC191670), SPCSV (AJ428554).

## Supporting Information

S1 Fig Sense-mediated silencing of *gfp* expression in the *gfp*-transgenic *C*. *elegans* strain RT476, which expresses *gfp* under the intestine-specific promoter *vha-6*. (**A**) Representative images of *C*. *elegans* intestine taken with bright field (BF) illumination to observe morphology or UV light to observe GFP fluorescence. *gfp* silencing was induced by feeding the animals an *E*. *coli* strain expressing *gfp* mRNA (sense GFP). Controls included feeding with bacteria harboring an empty (no insert) plasmid or a plasmid lacking the T7 promoter. (**B**) Comparison of the normalized average GFP fluorescence intensity 72 h post-feeding with bacteria in three independent experiments. Bars indicate S.E.M. (n = 23–34). GFP fluorescence in “sense GFP” treatment was significantly lower (unpaired t-test; p < 0.001) than in the two other treatments that did not differ from each other. (**C**) Detection of *gfp* mRNA in *E*. *coli* using strand-specific RT-PCR. Bacteria analyzed with the three primer pairs in lanes 1–3 were transformed with pET24b^+^ empty (no insert; a negative control), whereas bacteria analyzed in lanes 4–6 were transformed with pET24b^+^GFPopt for expression of *gfp* mRNA. The RNA samples analyzed were: lanes 1 and 4, *gfp* mRNA; lanes 2 and 5, RNA not subjected to reverse transcription; lanes 3 and 6, antisense *gfp* mRNA. The amplification product in lane 4 was expected and is of the expected size (~770 bp). L, 1-kb DNA marker ladder (Fermentas).
(PDF)

S2 Fig Enhanced accumulation of an RNAi-deficient *Tobacco rattle virus* (TRV) RNA1 replicon following co-expression with PPR3 in leaves of *Nicotiana benthamiana*. (**A**) Full-grown leaves of *N*. *benthamiana* were co-infiltrated with an *Agrobacterium tumefaciens* strain expressing PPR3 and a strain expressing the replicon TRV-M1 of *Tobacco rattle virus* (TRV) RNA1, which lacks the *16K* gene coding for an RNAi suppressor [76], or were infiltrated only with the *A*. *tumefaciens* strain expressing TRV-M1 (control). (**B**) Leaves of *N*. *benthamiana* were co-infiltrated with three *A*. *tumefaciens* strains expressing PPR3, TRV-M1 and TRV-RNA2(GFP), respectively, and compared with controls, i.e., agroinfiltration for co-expression of TRV-M1 and TRV-RNA2(GFP). Control agroinfiltration was done to the opposite site of the mid-rib in the same leaf. Samples from infiltrated leaf tissues were collected for analysis 3 days post-infiltration (d.p.i.), before appearance of necrotic symptoms at 4 d.p.i. TRV RNA1 amounts were estimated using quantitative reverse transcription realtime PCR, as described [76]. RNA1 amounts (fold change) in (**A**) and (**B**) are shown for three independently tested leaves relative to the mean of the controls (no PPR3) of the three leaves (= 1.0). Blue bars indicate TRV RNA1 accumulation in the presence of PPR3, whereas red bars (—) show accumulation of RNA1 in controls lacking PPR3. TRV RNA2 influences accumulation of RNA1 by an unknown mechanism [76], which explains the less consistent results in (B). However, in all experiments, there was a consistent tendency for enhanced accumulation of TRV RNA1 in the presence of PPR3.
(PDF)

S1 Table *C*. *elegans* strains used in silencing assays. (DOC)

S2 Table Primers used in the study. (DOC)
