# Supplementary material for: Suppression of RNAi by dsRNA-Degrading RNaseIII Enzymes of Viruses in Animals and Plants
Source: PLoS Pathog. 2015 Mar 6;11(3):e1004711. doi: 10.1371/journal.ppat.1004711 (PMC4352025; doi:10.1371/journal.ppat.1004711)
Supplement: S1 Fig — (A) Representative images of C. elegans intestine taken with bright field (BF) illumination to observe morphology or UV light to observe GFP fluorescence. gfp silencing was induced by feeding the animals an E. coli strain expressing gfp mRNA (sense GFP). Controls included feeding with bacteria harboring an empty (no insert) plasmid or a plasmid lacking the T7 promoter. (B) Comparison of the normalized average GFP fluorescence intensity 72 h post-feeding with bacteria in three independent experiments. Bars indicate S.E.M. (n = 23–34). GFP fluorescence in “sense GFP” treatment was significantly lower (unpaired t-test; p < 0.001) than in the two other treatments that did not differ from each other. (C) Detection of gfp mRNA in E. coli using strand-specific RT-PCR. Bacteria analyzed with the three primer pairs in lanes 1–3 were transformed with pET24b+ empty (no insert; a negative control), whereas bacteria analyzed in lanes 4–6 were transformed with pET24b+GFPopt for expression of gfp mRNA. The RNA samples analyzed were: lanes 1 and 4, gfp mRNA; lanes 2 and 5, RNA not subjected to reverse transcription; lanes 3 and 6, antisense gfp mRNA. The amplification product in lane 4 was expected and is of the expected size (~770 bp). L, 1-kb DNA marker ladder (Fermentas). (PDF) [file ppat.1004711.s001.pdf]

Supporting Information

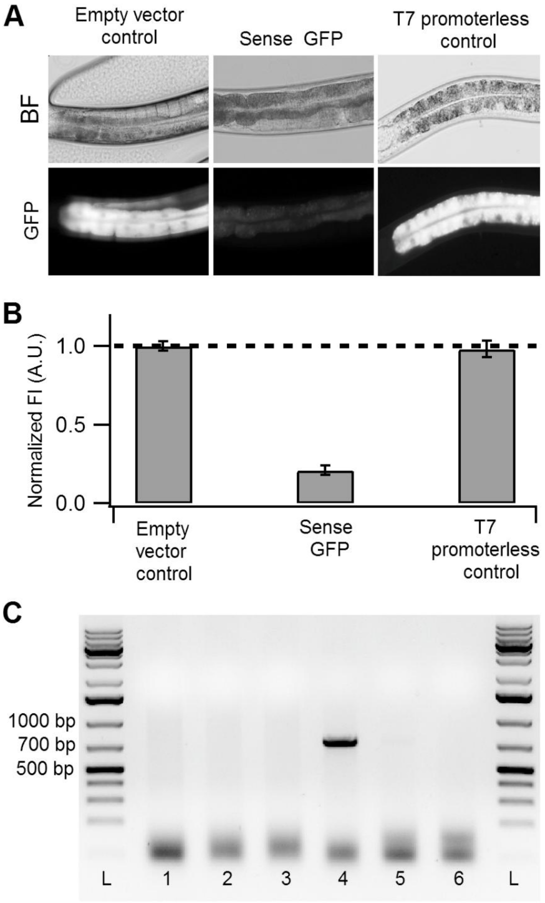

Figure S1. Sense-mediated silencing of *gfp* expression in the *gfp*-transgenic *C. elegans* strain RT476, which expresses *gfp* under the intestine-specific promoter *vha-6*. (**A**) Representative images of *C. elegans* intestine taken with bright field (BF) illumination to observe morphology or UV light to observe GFP fluorescence. *gfp* silencing was induced by feeding the animals an *E. coli* strain expressing *gfp* mRNA (sense GFP). Controls included feeding with bacteria harboring an empty (no insert) plasmid or a plasmid lacking the T7 promoter. (**B**) Comparison of the normalized average GFP fluorescence intensity 72 h post-feeding with bacteria in three independent experiments. Bars indicate S.E.M. (n = 23–34). GFP fluorescence in "sense GFP" treatment was significantly lower (unpaired t-test; $p < 0.001$) than in the two other treatments that did not differ from each other. (**C**) Detection of *gfp* mRNA in *E. coli* using strand-specific RT-PCR. Bacteria analyzed with the three primer pairs in lanes 1–3 were transformed with pET24b$^+$ empty (no insert; a negative control), whereas bacteria analyzed in lanes 4–6 were transformed with pET24b$^+$GFPopt for expression of *gfp* mRNA. The RNA samples analyzed were: lanes 1 and 4, *gfp* mRNA; lanes 2 and 5, RNA not subjected to reverse transcription; lanes 3 and 6, antisense *gfp* mRNA. The amplification product in lane 4 was expected and is of the expected size (~770 bp). L, 1-kb DNA marker ladder (Fermentas).
