# Supplementary material for: Suppression of RNAi by dsRNA-Degrading RNaseIII Enzymes of Viruses in Animals and Plants
Source: PLoS Pathog. 2015 Mar 6;11(3):e1004711. doi: 10.1371/journal.ppat.1004711 (PMC4352025; doi:10.1371/journal.ppat.1004711)
Supplement: S2 Fig — (A) Full-grown leaves of N. benthamiana were co-infiltrated with an Agrobacterium tumefaciens strain expressing PPR3 and a strain expressing the replicon TRV-M1 of Tobacco rattle virus (TRV) RNA1, which lacks the 16K gene coding for an RNAi suppressor [76], or were infiltrated only with the A. tumefaciens strain expressing TRV-M1 (control). (B) Leaves of N. benthamiana were co-infiltrated with three A. tumefaciens strains expressing PPR3, TRV-M1 and TRV-RNA2(GFP), respectively, and compared with controls, i.e., agroinfiltration for co-expression of TRV-M1 and TRV-RNA2(GFP). Control agroinfiltration was done to the opposite site of the mid-rib in the same leaf. Samples from infiltrated leaf tissues were collected for analysis 3 days post-infiltration (d.p.i.), before appearance of necrotic symptoms at 4 d.p.i. TRV RNA1 amounts were estimated using quantitative reverse transcription realtime PCR, as described [76]. RNA1 amounts (fold change) in (A) and (B) are shown for three independently tested leaves relative to the mean of the controls (no PPR3) of the three leaves (= 1.0). Blue bars indicate TRV RNA1 accumulation in the presence of PPR3, whereas red bars (—) show accumulation of RNA1 in controls lacking PPR3. TRV RNA2 influences accumulation of RNA1 by an unknown mechanism [76], which explains the less consistent results in (B). However, in all experiments, there was a consistent tendency for enhanced accumulation of TRV RNA1 in the presence of PPR3. (PDF) [file ppat.1004711.s002.pdf]

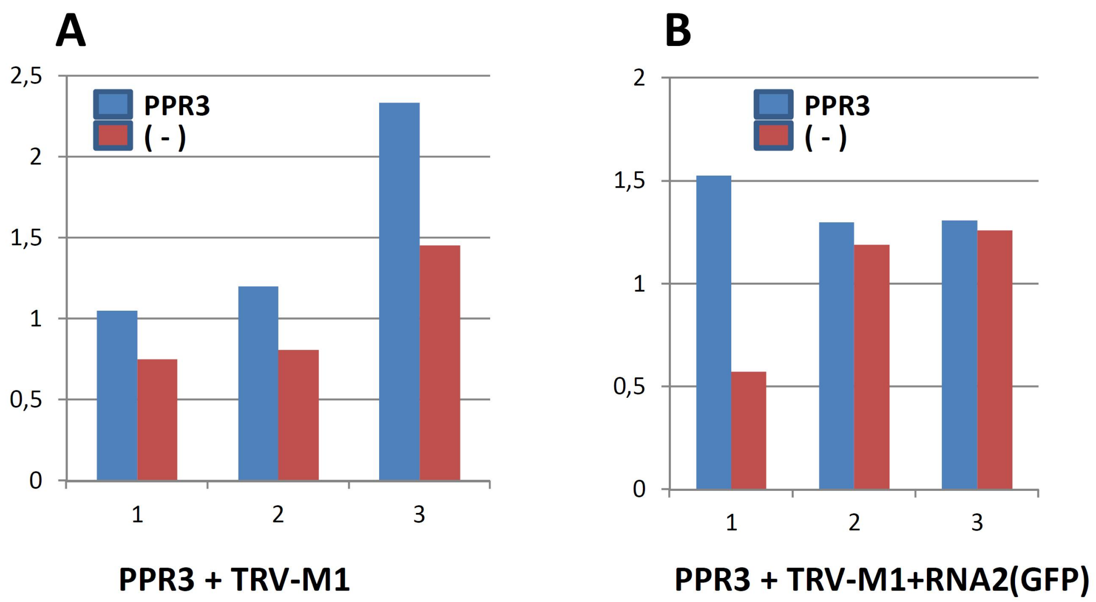

Figure S2. Enhanced accumulation of an RNAi-deficient *Tobacco rattle virus* (TRV) RNA1 replicon following co-expression with PPR3 in leaves of *Nicotiana benthamiana***.**
(**A**) Full-grown leaves of *N. benthamiana* were co-infiltrated with an *Agrobacterium tumefaciens* strain expressing PPR3 and a strain expressing the replicon TRV-M1 of *Tobacco rattle virus* (TRV) RNA1, which lacks the *16K* gene coding for an RNAi suppressor [76], or were infiltrated only with the *A. tumefaciens* strain expressing TRV-M1 (control). (**B**) Leaves of *N. benthamiana* were co-infiltrated with three *A. tumefaciens* strains expressing PPR3, TRV-M1 and TRV-RNA2(GFP), respectively, and compared with controls, i.e., agroinfiltration for co-expression of TRV-M1 and TRV-RNA2(GFP). Control agroinfiltration was done to the opposite site of the mid-rib in the same leaf. Samples from infiltrated leaf tissues were collected for analysis 3 days post-infiltration (d.p.i.), before appearance of necrotic symptoms at 4 d.p.i. TRV RNA1 amounts were estimated using quantitative reverse transcription realtime PCR, as described [76]. RNA1 amounts (fold change) in (**A)** and (**B)** are shown for three independently tested leaves relative to the mean of the controls (no PPR3) of the three leaves (= 1.0). Blue bars indicate TRV RNA1 accumulation in the presence of PPR3, whereas red bars (-) show accumulation of RNA1 in controls lacking PPR3. TRV RNA2 influences accumulation of RNA1 by an unknown mechanism [76], which explains the less consistent results in (B). However, in all experiments, there was a consistent tendency for enhanced accumulation of TRV RNA1 in the presence of PPR3.
