# Supplementary material for: Suppression of RNAi by dsRNA-Degrading RNaseIII Enzymes of Viruses in Animals and Plants
Source: PLoS Pathog. 2015 Mar 6;11(3):e1004711. doi: 10.1371/journal.ppat.1004711 (PMC4352025; doi:10.1371/journal.ppat.1004711)
Supplement: S1 Table — (DOC) [file ppat.1004711.s003.doc]

**Supporting Information**

**Table S1.** ***C. elegans* strains used in silencing assays^a^**

| **Strain/Genotype**^b^ | **Remark** | **Labeled Tissue** |
| --- | --- | --- |
| RT476/[pwIs170](http://legacy.wormbase.org/db/gene/transgene?name=pwIs170;class=Transgene)^1^ | Is[Pvha-6::GFP-RAB-7; Cbr-unc-119(+)] | Anterior intestine, posterior intestine |
| SJ4157/zcIs21^2^ | Is[Phsp-16::clpp-1(WT)::3xmyc-His-tag + Pmyo-3::GFP] | Body wall muscle |
| NM2415/jsIs682^3^ | Is[Prab-3::GFP::RAB-3; pJM23] | Nerve cord, nerve ring |
| NP738/cdIs36^4^ | Is[Pcc1::C31E10.7::GFP + unc-119(+) + Pmyo-2::GFP] | Head muscle |
| NL2099/rrf-3^5^ | pk1426: 3015-bp deletion | - |
| rrf-3; pwIs170 | NL2099 crossed with RT476 | Anterior intestine, posterior intestine |

^a^Detailed information on the strains and their attributes can be found at www.wormbase.org/ and http://www.cgc.cbs.umn.edu/

^b^The strains/genotypes of *C. elegans* were supplied by:

^1^ B Grant, Rutgers University, Piscataway, NJ

^2^ D Ron, Skirball Institute, New York, NY

^3^ M Nonet, Washington University, St. Louis, MO

^4^ J Fares, University of Arizona, Tucson, AZ

^5^ R Plasterk, Hubrecht Laboratory, Utrecht, The Netherlands
