# Supplementary material for: Suppression of RNAi by dsRNA-Degrading RNaseIII Enzymes of Viruses in Animals and Plants
Source: PLoS Pathog. 2015 Mar 6;11(3):e1004711. doi: 10.1371/journal.ppat.1004711 (PMC4352025; doi:10.1371/journal.ppat.1004711)
Supplement: S2 Table — (DOC) [file ppat.1004711.s004.doc]

**Supporting Information / Table S2. Primers used in the study**

| **Primer** | **Sequence (5’–3’)^a^** | **Application** |
| --- | --- | --- |
| PPR3 *Not*I fwd | aaggaaaaaagcggccgctatggaaggttggttgggaaacttgc | Amplification of *PPR3* from PPIV DNA for plant transformation and *E. coli* expression |
| PPR3 *Fse*I rev | tgactggccggccctatactcccttggcatgaacg | Cloning of PPR3 for *E. coli* expression |
| PPR3 *Fse*I +StrepII rev | tgactggccggccctaCTTTTCAAATTGCGGATGGGACCAtactcccttggcatgaacggtc | Amplification of *PPR3* from PPIV DNA for plant transformation, translational fusion with StrepII-tag |
| T7 GFP fwd | **taatacgactcactataggg**atgggaaaagggaggagttg | long dsRNA production, T7 promoter |
| Phi6 GFP rev | **ggaaaaaaa**tcaagcttctagagatccttcc | long dsRNA production, Phi6 promoter |
| PPR3-Ala fwd | gacgacaactacg*C*ggctctggagattgtggggg*C*cggagtggcgtc | Site-directed mutagenesis of *PPR3* |
| PPR3-Ala rev | gacgccactccg*G*cccccacaatctccagagcc*G*cgtagttgtcgtc | Site-directed mutagenesis of *PPR3* |
| pET GFPopt fwd | ccgctcgagatgggaaaagggaggagttg | RT-PCR for sense/antisense-GFP |
| pET GFPopt rev | ccggaattctcaagcttctagagatcttcc | RT-PCR for sense/antisense-GFP |
| 54_01 PPR3 *Xba*I fwd | ctagtctagaatggaaggttggttgggaaac | Cloning of *PPR3*, *PPR3-Ala* for *C. elegans* transformation |
| 54_01 PPR3 *Nhe*I rev | cggtagctagcttacccgggtactcccttggcatgaacggtc | Cloning of *PPR3*, *PPR3-Ala* for *C. elegans* transformation |
| 54_01 CSR3 *Xba*I fwd | ctagtctagaatggttccgatttattccgac | Cloning of *CSR3*, *CSR3-Ala* for *C. elegans* transformation |
| 54_01 CSR3 *Xma*I rev | ggggtaccttacccgggactcagatttagagcttcaac | Cloning of *CSR3*, *CSR3-Ala* for *C. elegans* transformation |
| 54_01 dTCSR3 *Xba*I fwd | ggctctagatggtgagcaagggcgaggagg | Amplification of *dTomato*, insertion in *C. elegans* vectors |
| 54_01 dTCSR3 *Xba*I rev | ggctctagacttgtacagctcgtccatgccg | Amplification of *dTomato*, insertion in *C. elegans* vectors |
| pMT_CSR3 *EcoR*I fwd | atagaattcagaatggttccgatttattccgac | Cloning of *CSR3*, *CSR3-Ala* for Drosophila transfection |
| pMT_CS3R3 *Xho*I rev | taactcgagactcagatttagagcttcaac | Cloning of *CSR3*, *CSR3-Ala* for Drosophila transfection |
| pMT_PPR3 *EcoR*I fwd | atagaattcagaatggaaggttggttgggaaacttg | Cloning of *PPR3*, *PPR3-Ala* for Drosophila transfection |
| pMT_PPR3 *Xho*I rev | taactcgagtactcccttggcatgaacgg | Cloning of *PPR3*, *PPR3-Ala* for Drosophila transfection |

^a^The restriction sites essential for cloning are underlined. StrepII-tag nucleotide sequence is denoted with capital letters. Bold letters indicate the recognition site for T7 polymerase (T7 GFP fwd) or Phi6 polymerase (Phi6 GFP rev). Nucleotide replacements denoted with capital/italic letters mark the introduced mutations.
